# Supplementary material for: Prefrontal coding of learned and inferred knowledge during REM and NREM sleep
Source: Nat Commun. 2024 Jun 24;15:4566. doi: 10.1038/s41467-024-48816-x (PMC11196720; doi:10.1038/s41467-024-48816-x)
Supplement: Supplementary file 1 — Supplementary Information [file 41467_2024_48816_MOESM1_ESM.pdf]

Supplementary Information for

Prefrontal coding of learned and inferred knowledge during REM and NREM sleep

Kareem Abdou[1,2,3,4], Masanori Nomoto[1,2,4,5], Mohamed H. Aly[1,2,4,6], Ahmed Z. Ibrahim[1,2,3,4], Kiriko Choko[1,2,4], Reiko Okubo-Suzuki[1,2,4], Shin-ichi Muramatsu[7,8], Kaoru Inokuchi[1,2,4*]

Affiliations

[1]Research Centre for Idling Brain Science, University of Toyama, Toyama, 930-0194, Japan

[2]Department of Biochemistry, Graduate School of Medicine and Pharmaceutical Sciences, University of Toyama, Toyama, Japan

[3]Department of Biochemistry, Faculty of Pharmacy, Cairo University, Cairo 11562, Egypt

[4]CREST, Japan Science and Technology Agency (JST), University of Toyama, Toyama, Japan

[5]Japan Agency for Medical Research and Development (AMED), Tokyo, Japan

[6]Pharmacology Department, Faculty of Pharmacy, The British University in Egypt, Cairo 11837, Egypt

[7]Division of Neurological Gene Therapy, Centre for Open Innovation, Jichi Medical University, Tochigi 3290498, Japan

[8]Centre for Gene and Cell Therapy, The Institute of Medical Science, The University of Tokyo, Tokyo 1088639, Japan

[*]Correspondence should be addressed to K.I.: inokuchi@med.u-toyama.ac.jp

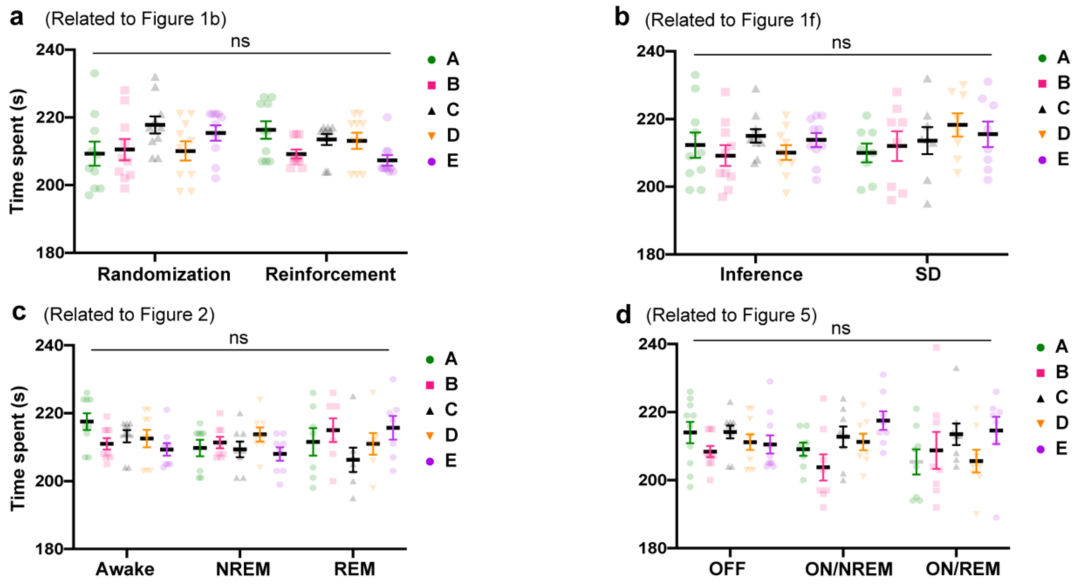

Supplementary Fig. 1 | Mice had no innate preference to any context during habituation. a-d, Time spent in each context during the habituation phase. The number of animals in each panel is mentioned in the corresponding Figure legend. SD, sleep deprivation; NREM, non-rapid eye movement; REM, rapid eye movement. Statistical comparisons were made using a two-way repeated-measures analysis of variance (ANOVA) with Tukey's multiple comparison test (**a-d**). ns, not significant ($P > 0.05$). Data are presented as the mean ± standard error of the mean (s.e.m.). Source data are provided as a Source Data file. Detailed statistics are shown in Supplementary Data 1.

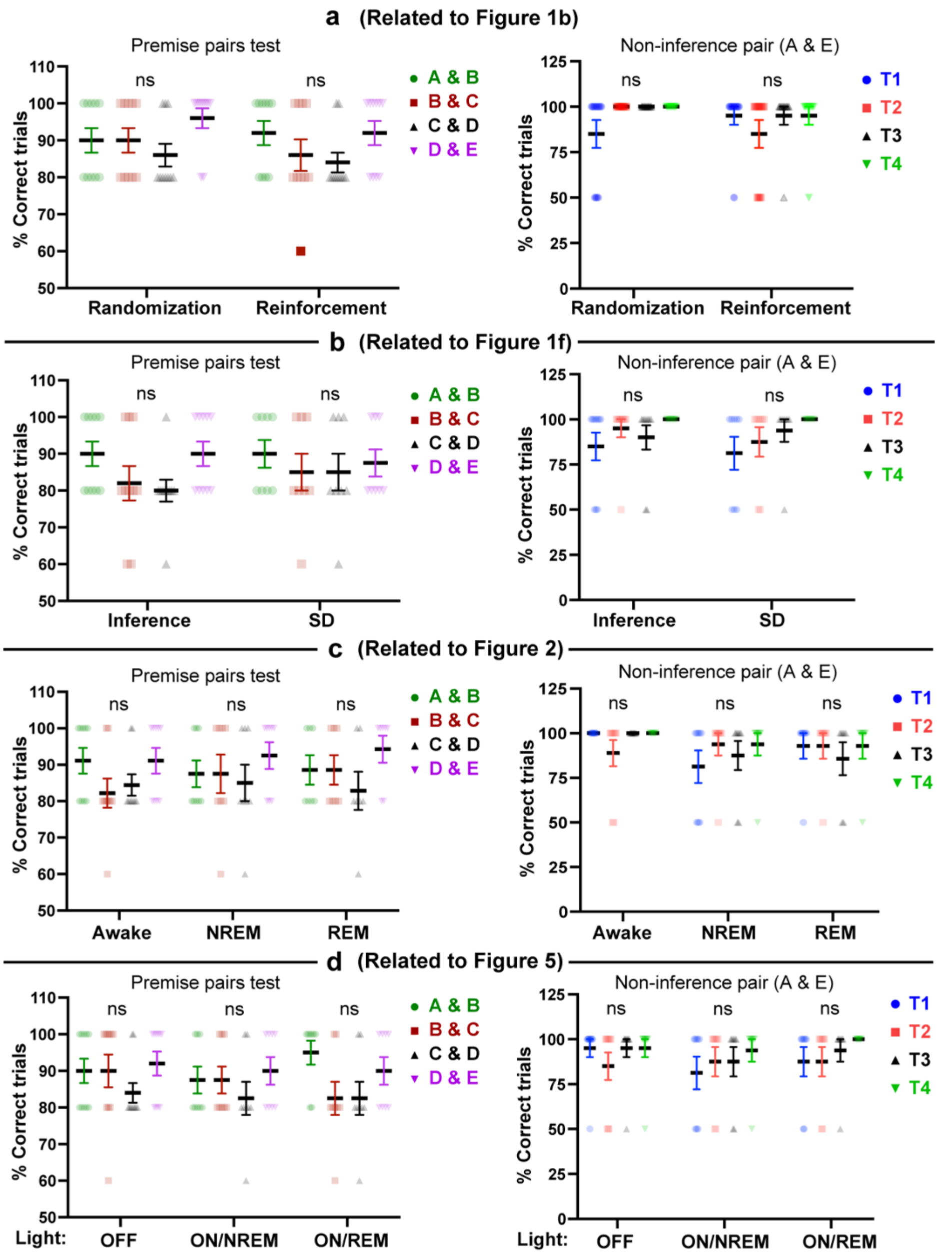

Supplementary Fig. 2 | Correct performance during testing original premise pairs and non-inference pair. a-d, Performance during test sessions; the percent of correct trials during the original premise pairs test (left) and during the non-inference test (right). T1, test 1; T2, test 2; T3, test 3; T4, test 4. SD, sleep deprivation; NREM, non-rapid eye movement; REM, rapid eye movement. The number of animals in each panel is mentioned in the corresponding Figure legend. Statistical comparisons were made using a two-way repeated-measures analysis of variance (ANOVA) with Tukey's multiple comparison test (**a-d**). ns, not significant (*P* > 0.05).

Data are presented as the mean ± standard error of the mean (s.e.m.). Source data are provided as a Source Data file. Detailed statistics are shown in Supplementary Data 1.

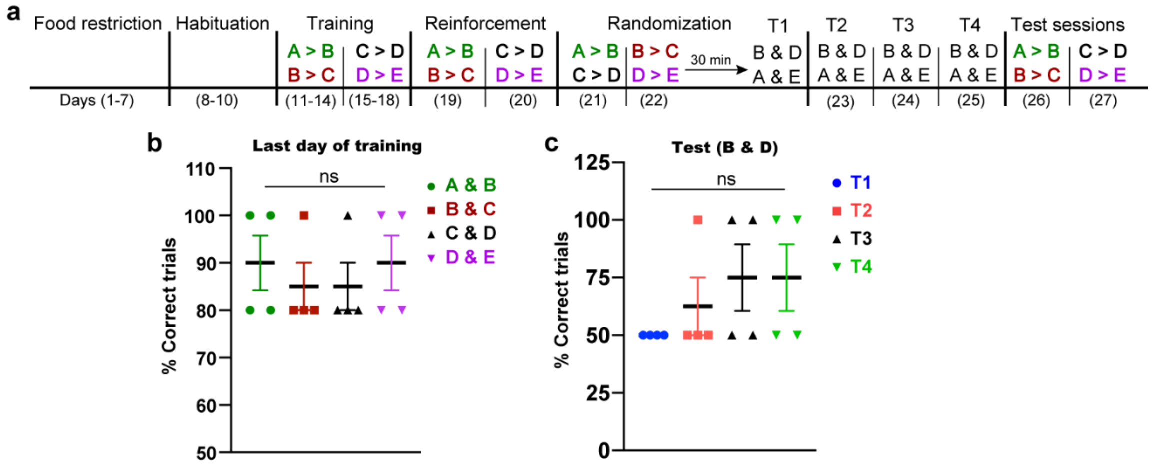

Supplementary Fig. 3 | Complete randomization is necessary for inference emergence. a, The behavioral schedule used in Figure 1, but without complete randomization which requires 4 days to ensure full interaction between all premise pairs. The order of presentation of the premise pairs during the randomization stage is different across animals within the same group (See Methods; Table 1). **b**, Performance during the last day of training (day 14 for A > B and B > C; day 18 for C > D and D > E) for each premise pair was calculated as the percent of correct trials out of the total number of trials. **c,** Performance during test sessions; the percent of correct trials during the inference test sessions. Statistical comparisons were made using a one-way repeated-measures analysis of variance (ANOVA) with Tukey's multiple comparison test; *n*=4 mice; ns, not significant ($P > 0.05$). Data are presented as the mean ± standard error of the mean (s.e.m.). Source data are provided as a Source Data file. Detailed statistics are shown in Supplementary Data 1.

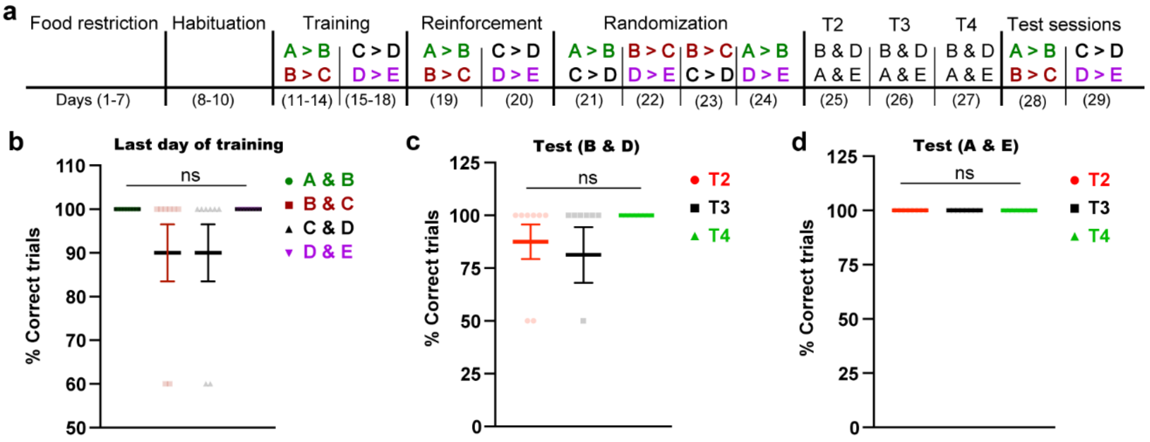

Supplementary Fig. 4 | Inferential behavior does not require priming by preexposure to the testing environment. a, The behavioral schedule used in Figure 1, but without exposure to T1 session. The order of presentation of the premise pairs during the randomization stage is different across animals within the same group (See Methods; Table 1). **b**, The performance during the last day of training (day 14 for A > B and B > C; day 18 for C > D and D > E) for each premise pair was calculated as the percent of correct trials out of the total number of trials. **c-d,** Performance during test sessions; the percent of correct trials during the inference test (**c**) and during the non-inference test (**d**). Statistical comparisons were made using a one-way repeated-measures analysis of variance (ANOVA) with Tukey's multiple comparison test; n=8 mice; ns, not significant ($P > 0.05$). Data are presented as the mean ± standard error of the mean (s.e.m.). The Experiment was independently repeated two times. Source data are provided as a Source Data file. Detailed statistics are shown in Supplementary Data 1.

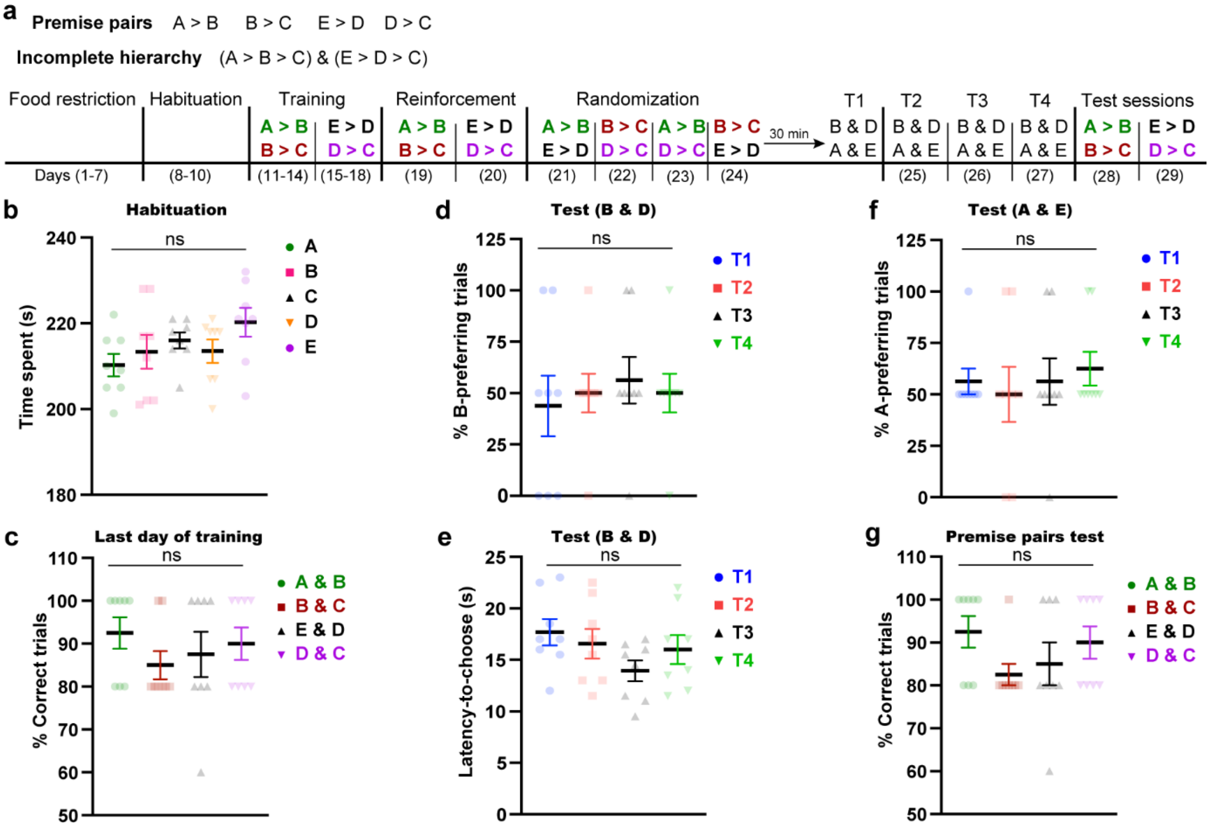

Supplementary Fig. 5 | Without the complete hierarchy, mice don't have any preference to context B or context D. a, The behavioral schedule used to test the impact of the complete hierarchy on inference development. **b**, Time spent in each context during the habituation phase. **c,** The performance during the last day of training (day 14 for A > B and B > C; day 18 for E > D and D > C) for each premise pair was calculated as the percent of correct trials out of the total number of trials. **d-g,** Performance during test sessions; the percent of B-preferring trials (**d**), the latency time to choose during the test (**e**), the percent of A-preferring trials (**f**), the percent of correct trials during the original premise pairs test (**g**). T1, test 1; T2, test 2; T3, test 3; T4, test 4. $n = 8$ mice. Statistical comparisons were made using a one-way repeated-measures analysis of variance (ANOVA) with Tukey's multiple comparison test (**b-g**). ns, not significant ($P > 0.05$). Data are presented as the mean ± standard error of the mean (s.e.m.). The Experiment was independently repeated two times. Source data are provided as a Source Data file. Detailed statistics are shown in Supplementary Data 1.

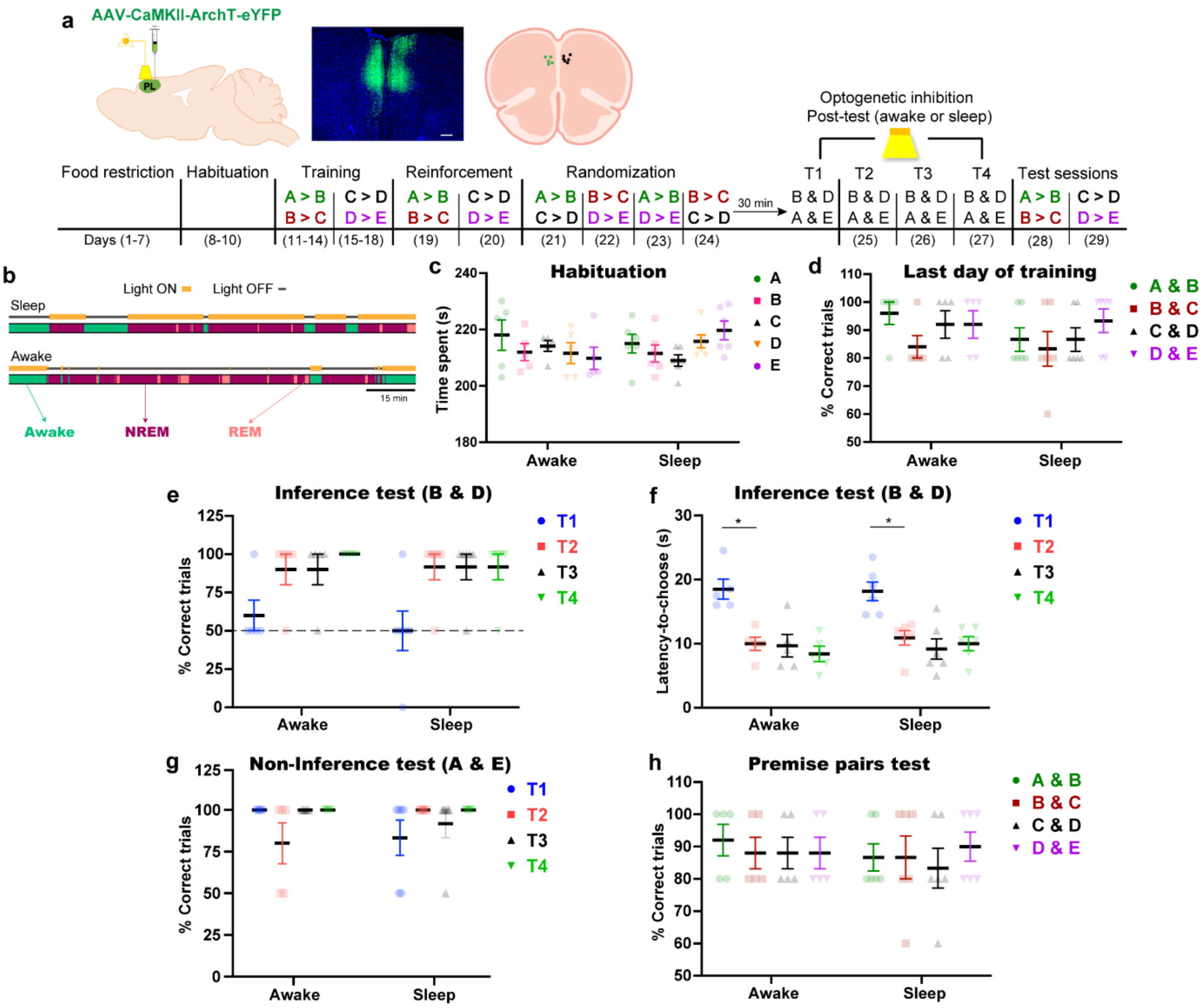

Supplementary Fig. 6 | Prelimbic cortex activity is not required for the emergence of inference. a, Top, Labelling of excitatory neurons of the prelimbic cortex (PL) with ArchT (left), coronal section from the mouse brain showing the expression of ArchT-eYFP (green) in the PL (middle) and optic fiber tip location (right). Black dots, mice in the awake group; Green dots, mice in the sleep group. Blue, 4′,6-diamidino-2-phenylindole (DAPI) staining. Scale bar, 100 µm. Bottom, The behavioral schedule used to manipulate PL activity during sleep or awake periods after test sessions. The order of presentation of the premise pairs during the randomization stage is different across animals within the same group (See Methods; Table 1). **b**, Diagram showing the state-specific manipulation. Scale bar, 15 minutes. **c,** Time spent in each context during the habituation phase**. d,** Performance during the last day of training (day 14 for A > B and B > C; day 18 for C > D and D > E) for each premise pair. **e-h,** Performance

during the test sessions; the percent of correct trials during the inference test (**e**), latency time to choose during the inference test (**f**), during the non-inference test (**g**), during the original premise pairs test (**h**). T1, test 1; T2, test 2; T3, test 3; T4, test 4. $n = 5$ mice for the awake group; $n = 6$ mice for the sleep group. Statistical comparisons were made using a two-way repeated-measures analysis of variance (ANOVA) with Tukey's multiple comparison test (**c-h**). In the non-inference test (**g**), the statistical significance denotes the comparison between performance of the two groups relative to the chance level (50%). $^{*}P < 0.05$; $^{****}P < 0.0001$; ns, not significant ($P > 0.05$). Data are presented as the mean ± standard error of the mean (s.e.m.). The Experiment was independently repeated three times. Source data are provided as a Source Data file. Detailed statistics are shown in Supplementary Data 1.

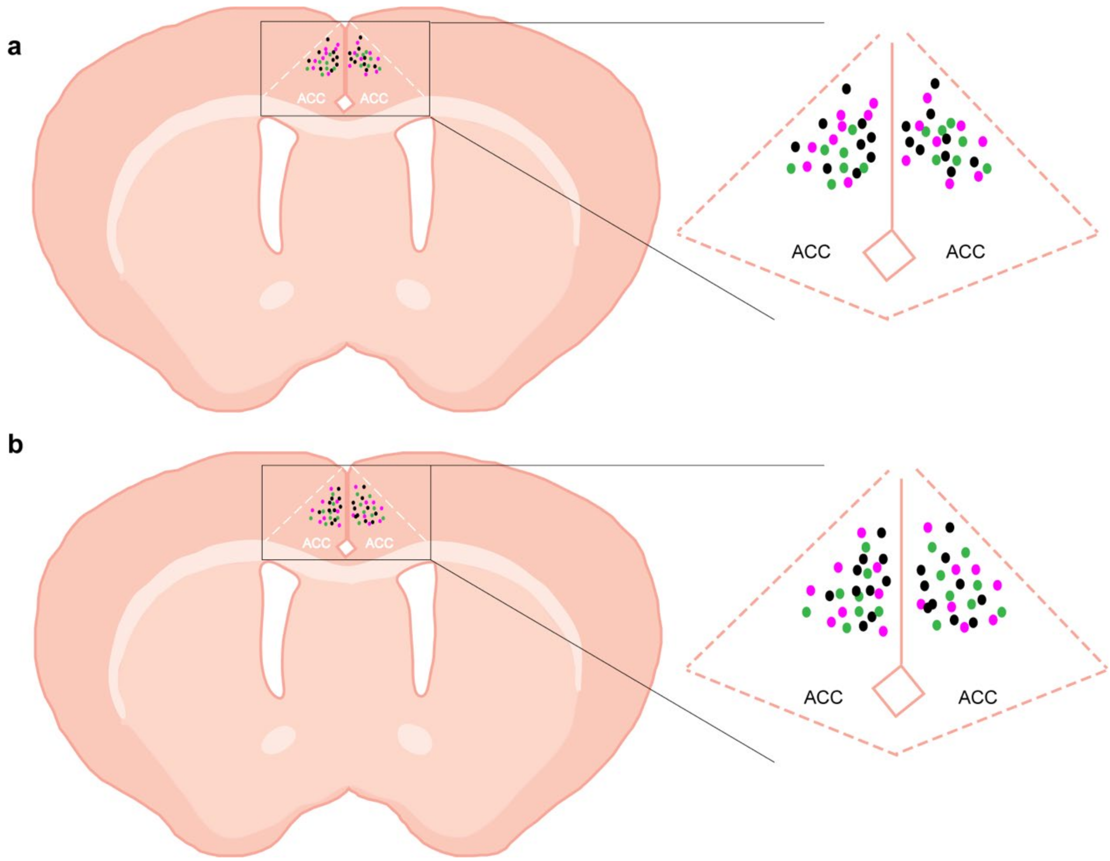

Supplementary Fig. 7 | Optic fiber locations in mice used in the manipulation experiments. **a**, Coronal section from the mouse brain showing the optic fiber tip location for mice shown in Fig. 2. Black dots, mice in the awake group; Green dots, mice in the NREM group; Pink dots, mice in the REM group. **b**, Coronal section from the mouse brain showing the optic fiber tip location for mice shown in Fig. 5. Black dots, mice in the light off group; Green dots, mice in the NREM group; Pink dots, mice in the REM group. The anterior cingulate cortex (ACC) is indicated by dashed lines.

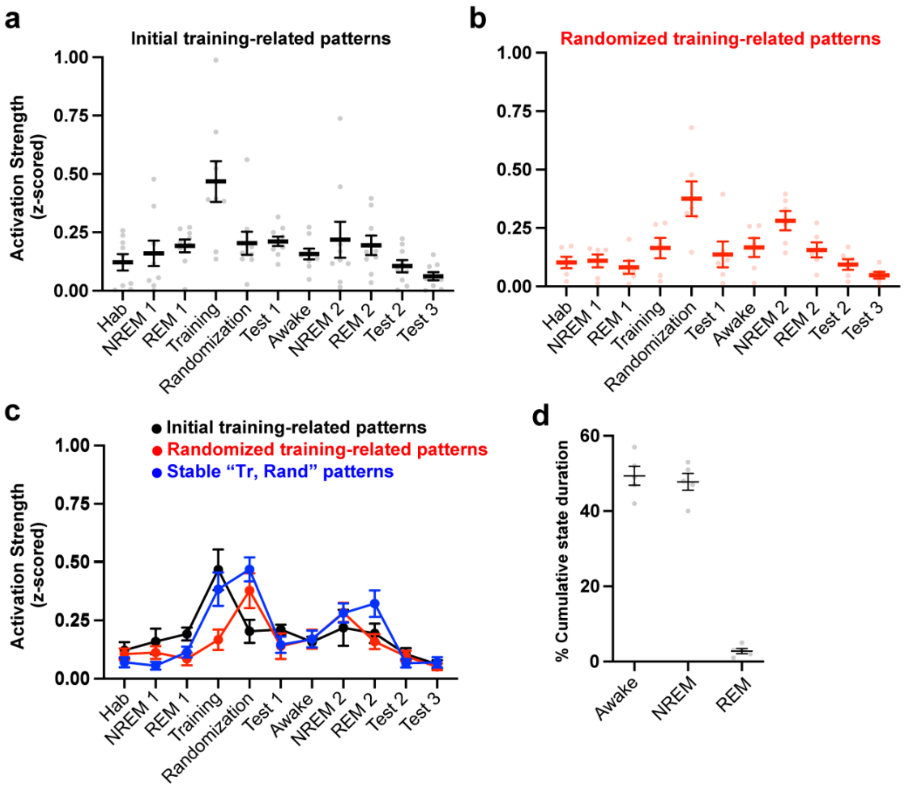

Supplementary Fig. 8 | Temporally different patterns representing the original premise pairs. a-c, Activation strength (z-scored) of the patterns representing the original premise pairs during initial training phase (**a, c**), during randomized training phase (**b, c**); *n* = 9 Initial training-related patterns (**a, c**), *n* = 6 Randomized training-related patterns (**b, c)**, *n* = 10 Stable training-related patterns (**c**). **d,** Cumulative duration of each state (awake and sleep states) in all 5 mice. Statistical comparison was done using one-way repeated-measures analysis of variance (ANOVA) (**a, b, d**) with Dunnett’s (**a, b**) and Tukey’s (**d**) multiple comparison tests; using two-way repeated-measures ANOVA with Tukey’s multiple comparison test (**c**). Data are presented as the mean ± standard error of the mean (s.e.m.). Source data are provided as a Source Data file. Detailed statistics are shown in Supplementary Data 1.

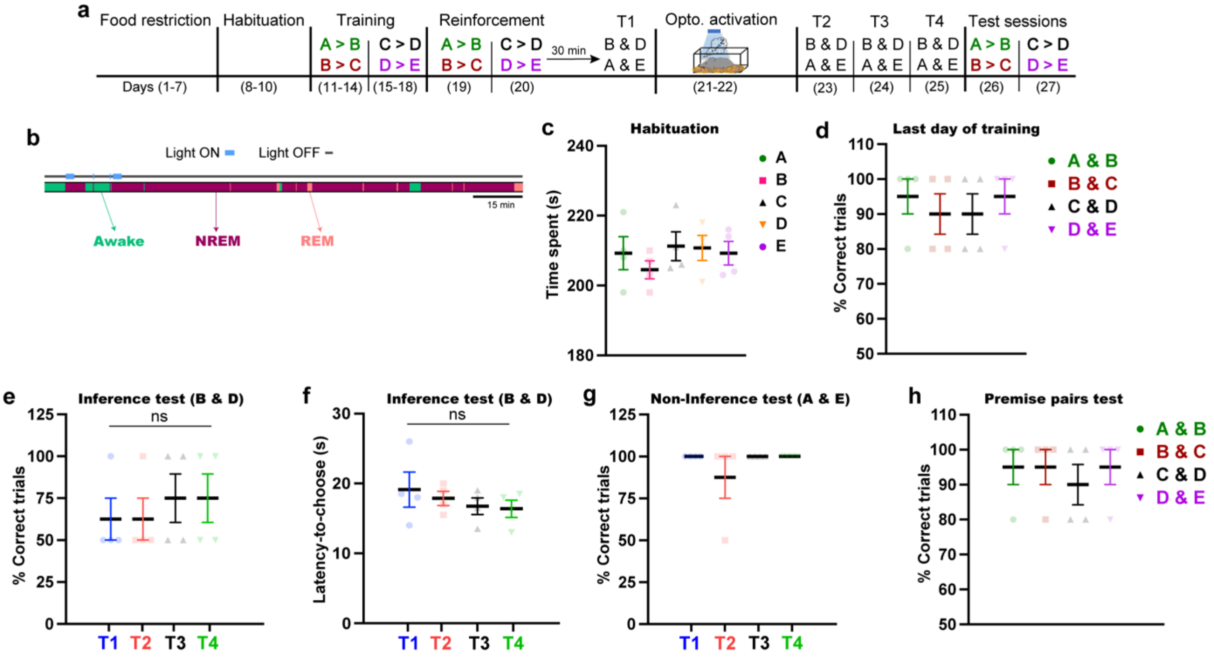

Supplementary Fig. 9 | Optogenetic activation of MEC→ACC circuit during NREM sleep is not sufficient for the emergence of inference. a, The behavioral schedule used to manipulate MEC→ACC circuit activity during NREM sleep after test sessions. **b**, Diagram showing the NREM-specific manipulation. The manipulation protocol mimicked the protocol used during REM sleep manipulation in Fig. 5 (see Methods) to avoid the prolonged light stimulation which might affect the results. Scale bar, 15 minutes. **c**, The time spent in each context during the habituation phase. **d**, Performance during the last day of training (day 14 for A > B and B > C; day 18 for C > D and D > E) for each premise pair. **e-h**, Performance during the test sessions; the percent of correct trials during the inference test (**e**), latency time to choose during the inference test (**f**), during the non-inference test (**g**), during the original premise pairs test (**h**). T1, test 1; T2, test 2; T3, test 3; T4, test 4; *n* = 4 mice. Statistical comparisons were made using a one-way repeated-measures analysis of variance (ANOVA) with Tukey's multiple comparison test (**c-h**). ns, not significant ($P > 0.05$). Data are presented as the mean ± standard error of the mean (s.e.m.). The Experiment was independently repeated two times. Source data are provided as a Source Data file. Detailed statistics are shown in Supplementary Data 1.

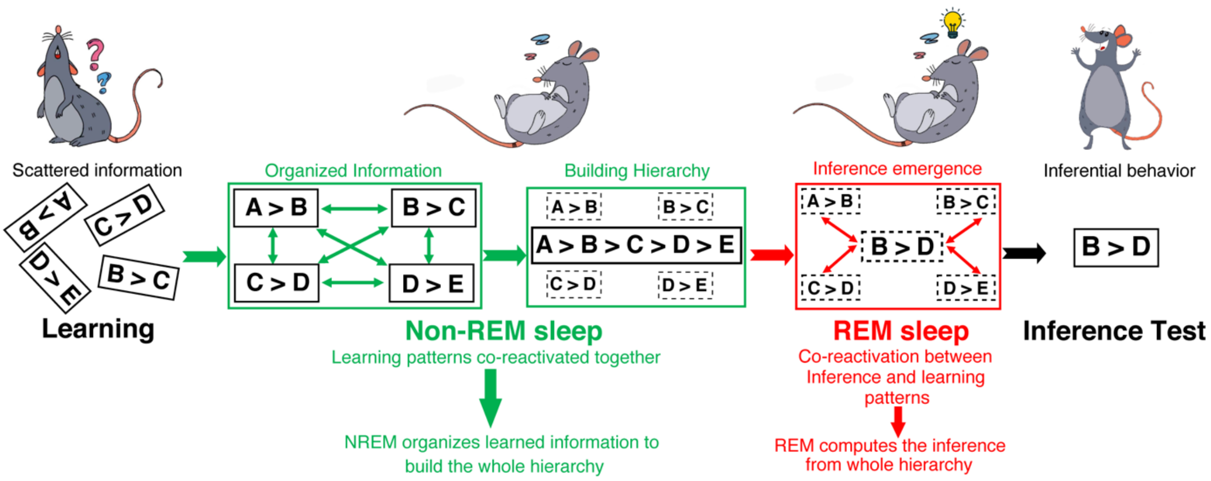

Supplementary Fig. 10 | A model of the coordinated roles of NREM & REM sleep in building inferential knowledge. Summary of the process of inference emergence. It starts with learning temporally distinct, but related information. Then, systematic organization of learned information in hierarchy during NREM sleep. Afterwards, building the inferential information from the hierarchy during REM sleep. Subsequently, inferential behavior appears in the test session. An arrow with arrowheads at both ends indicates synchronous activity.
