# Supplementary material for: Prefrontal coding of learned and inferred knowledge during REM and NREM sleep
Source: Nat Commun. 2024 Jun 24;15:4566. doi: 10.1038/s41467-024-48816-x (PMC11196720; doi:10.1038/s41467-024-48816-x)
Supplement: Supplementary file 3 — Description of additional supplementary files [file 41467_2024_48816_MOESM3_ESM.pdf]

Description of Additional Supplementary Files

Supplementary Data 1 - Statistical analysis details in main and supplementary figures.

Supplementary Movie 1 - Premise training

Supplementary Movie 2 - Inference test
