# Supplementary material for: Prefrontal coding of learned and inferred knowledge during REM and NREM sleep
Source: Nat Commun. 2024 Jun 24;15:4566. doi: 10.1038/s41467-024-48816-x (PMC11196720; doi:10.1038/s41467-024-48816-x)
Supplement: Supplementary file 7 — Reporting Summary [file 41467_2024_48816_MOESM7_ESM.pdf]

nature portfolio

Corresponding author(s): Kaoru Inokuchi

Last updated by author(s): Apr 5, 2024

Reporting Summary

Nature Portfolio wishes to improve the reproducibility of the work that we publish. This form provides structure for consistency and transparency in reporting. For further information on Nature Portfolio policies, see our Editorial Policies and the Editorial Policy Checklist.

Statistics

For all statistical analyses, confirm that the following items are present in the figure legend, table legend, main text, or Methods section.

n/a	Confirmed	
☐	☒	The exact sample size (*n*) for each experimental group/condition, given as a discrete number and unit of measurement
☐	☒	A statement on whether measurements were taken from distinct samples or whether the same sample was measured repeatedly
☐	☒	The statistical test(s) used AND whether they are one- or two-sided<br>*Only common tests should be described solely by name; describe more complex techniques in the Methods section.*
☐	☒	A description of all covariates tested
☐	☒	A description of any assumptions or corrections, such as tests of normality and adjustment for multiple comparisons
☐	☒	A full description of the statistical parameters including central tendency (e.g. means) or other basic estimates (e.g. regression coefficient) AND variation (e.g. standard deviation) or associated estimates of uncertainty (e.g. confidence intervals)
☐	☒	For null hypothesis testing, the test statistic (e.g. F, t, r) with confidence intervals, effect sizes, degrees of freedom and P value noted<br>*Give P values as exact values whenever suitable.*
☒	☐	For Bayesian analysis, information on the choice of priors and Markov chain Monte Carlo settings
☒	☐	For hierarchical and complex designs, identification of the appropriate level for tests and full reporting of outcomes
☒	☐	Estimates of effect sizes (e.g. Cohen's *d*, Pearson's *r*), indicating how they were calculated

Our web collection on statistics for biologists contains articles on many of the points above.

Software and code

Policy information about availability of computer code

Data collection	Behavioural data were captured with an overhead web camera (Logitech HD pro C920) mounted on a vertical stand. All electroencephalography & electromyograph recordings were done using OpenEx Software Suite v2.32 (RX8-2, Tucker Davis Technologies, USA) as mentioned and referenced in the "Methods" section. In vivo calcium imaging data were collected with nVista acquisition software (Inscopix, USA).
Data analysis	Statistical analyses were performed using Prism 8 (GraphPad Software, San Diego, CA, USA).<br>In vivo calcium imaging data were analyzed with Inscopix data processing software (IDPS v1.9.4.3801, Inscopix, USA), Inscopix Mosaic software (Mosaic v1.2.0, Inscopix, USA), Fiji software (version 1.53q, NIH, USA), HOTARU (version 3.3.2, see methods) and MATLAB 2020b (Mathworks, USA) with custom-made codes (https://github.com/IdlingBrainUT/Abdou2024_NatureCommunications).

For manuscripts utilizing custom algorithms or software that are central to the research but not yet described in published literature, software must be made available to editors and reviewers. We strongly encourage code deposition in a community repository (e.g. GitHub). See the Nature Portfolio guidelines for submitting code & software for further information.

Data

Policy information about availability of data

All manuscripts must include a data availability statement. This statement should provide the following information, where applicable:

- Accession codes, unique identifiers, or web links for publicly available datasets
- A description of any restrictions on data availability
- For clinical datasets or third party data, please ensure that the statement adheres to our policy

Source data are provided with this paper.
All data and resources that supported the findings of this study are available upon request. The datasets supporting this study will be deposited to a public repository when the ongoing studies using the same dataset are published.

Research involving human participants, their data, or biological material

Policy information about studies with human participants or human data. See also policy information about sex, gender (identity/presentation), and sexual orientation and race, ethnicity and racism.

Reporting on sex and gender	N/A
Reporting on race, ethnicity, or other socially relevant groupings	N/A
Population characteristics	N/A
Recruitment	N/A
Ethics oversight	N/A

Note that full information on the approval of the study protocol must also be provided in the manuscript.

Field-specific reporting

Please select the one below that is the best fit for your research. If you are not sure, read the appropriate sections before making your selection.

☒ Life sciences ☐ Behavioural & social sciences ☐ Ecological, evolutionary & environmental sciences

For a reference copy of the document with all sections, see nature.com/documents/nr-reporting-summary-flat.pdf

Life sciences study design

All studies must disclose on these points even when the disclosure is negative.

Sample size	No statistical method was used to pre-determine sample sizes. We determined sample sizes based on the previous literature (Nomoto et al., Nature Communications, 13:7413, 2022). The number of animals in each experimental group are standard sample sizes in this field.
Data exclusions	No data were excluded from the analysis.
Replication	All the experiments were replicated in multiple runs for at least two times.
Randomization	Animals were randomly allocated into different experimental groups.
Blinding	The experimenter was not blinded in this study because (1) all behavior data were video-recorded for future analysis by a blind researcher; (2) Mice performance was live captured without further processing. However, all data were reanalyzed by a researcher who is blind to the experimental conditions.

Reporting for specific materials, systems and methods

We require information from authors about some types of materials, experimental systems and methods used in many studies. Here, indicate whether each material, system or method listed is relevant to your study. If you are not sure if a list item applies to your research, read the appropriate section before selecting a response.

Materials & experimental systems

n/a	Involved in the study
	☒ Antibodies
	☒ Eukaryotic cell lines
☒	Palaeontology and archaeology
	☒ Animals and other organisms
☒	Clinical data
☒	Dual use research of concern
☒	Plants

Methods

n/a	Involved in the study
☒	ChIP-seq
☒	Flow cytometry
☒	MRI-based neuroimaging

Antibodies

Antibodies used	Rat anti-GFP (04404-84, GF090R, Nacalai Tesque Inc., Japan) & Donkey anti-rat IgG Alexa Fluor 488 (A21208, Molecular Probes).
Validation	We described all antibodies in the "Methods" section under the "Histology" part.<br>All antibodies are commercially available. Validation of all antibodies was performed by the manufacturers and is available through individual company websites as listed below:<br><br>Rat anti-GFP: https://www.e-nacalai.jp/ec2/EC-srchdetl.cfm?jump=EC-srchdetl&syohin=0440484&syubetsu=3&l=EN&lc=1<br><br>Donkey anti-rat IgG Alexa Fluor 488: https://www.thermofisher.com/antibody/product/Donkey-anti-Rat-IgG-H-L-Highly-Cross-Adsorbed-Secondary-Antibody-Polyclonal/A-21208

Eukaryotic cell lines

Policy information about cell lines and Sex and Gender in Research

Cell line source(s)	HEK293 T cells (240073, Agilent Tech, CA, USA).
Authentication	No further authentication was done for cell lines.
Mycoplasma contamination	Testing for Mycoplasma contamination was negative.
Commonly misidentified lines (See ICLAC register)	No commonly misidentified lines.

Animals and other research organisms

Policy information about studies involving animals; ARRIVE guidelines recommended for reporting animal research, and Sex and Gender in Research

Laboratory animals	Male C57BL/6J mice were purchased from Sankyo Labo Service Co. Inc. (Tokyo, Japan). Mice were (10-14) weeks old.
Wild animals	The study did not involve wild animals.
Reporting on sex	We used only male mice that were purchased from Sankyo Labo Service Co. Inc. (Tokyo, Japan).
Field-collected samples	The study did not include samples collected from the field.
Ethics oversight	All animals procedures were approved by 'The Animal Care and Use Committee' of the University of Toyama with approval number (A2022MED-9).

Note that full information on the approval of the study protocol must also be provided in the manuscript.
